# Cross-View Neuroimage Pattern Analysis in Alzheimer's Disease Staging

**DOI:** 10.3389/fnagi.2016.00023

**Published:** 2016-02-23

**Authors:** Sidong Liu, Weidong Cai, Sonia Pujol, Ron Kikinis, Dagan D. Feng, the Alzheimer's Disease Neuroimaging Initiative

**Affiliations:** ^1^The Biomedical and Multimedia Information Technology Research Group, School of Information Technologies, The University of SydneySydney, NSW, Australia; ^2^The Surgical Planning Laboratory, Harvard Medical School, Brigham and Women's HospitalBoston, MA, USA; ^3^The Med-X Research Institute, Shanghai Jiao Tong UniversityShanghai, China

**Keywords:** pattern recognition, neuroimaging, multi-modal, Alzheimer's disease, mild cognitive impairment

## Abstract

The research on staging of pre-symptomatic and prodromal phase of neurological disorders, e.g., Alzheimer's disease (AD), is essential for prevention of dementia. New strategies for AD staging with a focus on early detection, are demanded to optimize potential efficacy of disease-modifying therapies that can halt or slow the disease progression. Recently, neuroimaging are increasingly used as additional research-based markers to detect AD onset and predict conversion of MCI and normal control (NC) to AD. Researchers have proposed a variety of neuroimaging biomarkers to characterize the patterns of the pathology of AD and MCI, and suggested that multi-view neuroimaging biomarkers could lead to better performance than single-view biomarkers in AD staging. However, it is still unclear what leads to such synergy and how to preserve or maximize. In an attempt to answer these questions, we proposed a cross-view pattern analysis framework for investigating the synergy between different neuroimaging biomarkers. We quantitatively analyzed nine types of biomarkers derived from FDG-PET and T1-MRI, and evaluated their performance in a task of classifying AD, MCI, and NC subjects obtained from the ADNI baseline cohort. The experiment results showed that these biomarkers could depict the pathology of AD from different perspectives, and output distinct patterns that are significantly associated with the disease progression. Most importantly, we found that these features could be separated into clusters, each depicting a particular aspect; and the inter-cluster features could always achieve better performance than the intra-cluster features in AD staging.

## 1. Introduction

Alzheimers disease (AD) is the most common neurodegenerative disorder among aging people, which accounts for nearly 70% of all dementia cases. The symptoms of cognitive impairment develop gradually over years, and eventually lead to death (Kalaria et al., 2008). Currently, there is no cure for AD. The early signs of AD include a noticeable and measurable decline in memory, language, thinking, and other cognitive abilities. Patients with these symptoms are usually diagnosed as the Mild Cognitive Impairment (MCI). MCI does not notably interfere with daily activities, but those with MCI have a higher risk of later progressing to AD or other forms of dementia (Dubois and Albert, 2004; Jicha et al., 2006; Nettiksimmons et al., 2014). Many medical interventions may only be effective in the early course of the disease (Bond et al., 2012). Therefore, accurate staging of the disease, especially the detection of MCI, could help the physicians to identify the subjects at higher risk of developing dementia and allow the patients to receive early medical interventions before irreversible brain damages are formed.

Numerous biochemical and genetic biomarkers, e.g., increased cerebrospinal fluid (CSF) tau, phosphorylated tau and ubiquitin levels, low CSF Amyloid-β (*Aβ*_42_) concentration, and apolipoprotein E (ApoE) ϵ4 allele, have been proposed to detect AD onset and predict conversion of MCI and normal control (NC) to AD with high specificity and sensitivity (Trojanowski et al., 2010; Kandimalla et al., 2011, 2013, 2014; Andreasson et al., 2014). Recently, neuroimaging biomarkers have been increasingly used as additional markers for assessing the likelihood of such detection and prediction, since they can detect the changes in brain structure (e.g., atrophy) and function (e.g., hypometabolism, amyloid plaque, and neurofibrillary tangles formation) before the cognitive impairment symptoms appear (Perrin et al., 2009; Davatzikos et al., 2011; Ewers et al., 2011a,b; Hinrichs et al., 2011; Singh et al., 2012; Jacobs et al., 2015). Several large multi-modal neuroimaging data repositories, such as the Alzheimers Disease Neuroimaging Initiatives (ADNI) (Jack et al., 2008; Jagust et al., 2010) and Australian Imaging, Biomarker and Lifestyle Flagship Study of Aging (AIBL) (Sona et al., 2012), have been founded to facilitate the neuroimaging research in AD and MCI.

A variety of quantitative measures can be extracted from the neuroimaging data as biomarkers in the evaluation of AD and MCI patients, such as hippocampal volume loss (Schuff et al., 2009), ventricular boundary shift integral (Freeborough and Fox, 1997) extracted from structural MRI, and z-score (Minoshima et al., 1995) and t-map (Cai et al., 2010) extracted from FDG-PET. We refer to same type of features as a “view.” The terms, “view” and “modality,” are often used interchangeably in the computer vision community, but not in the medical imaging community. A modality, in medical imaging domain, usually means the image acquisition technique or scanning protocol, such as Magnetic Resonance Imaging (MRI), Positron Emission Tomography (PET), Computed Tomography (CT), Ultrasound, Single Photon Emission Computed Tomography (SPECT), functional MRI (fMRI), and Diffusion Tensor Imaging (DTI). However, a view means a specific type of measure extracted from a modality. Therefore, a modality may contain multiple views, but a view pertains to one modality.

MRI and PET are the two most widely used neuroimaging modalities in visualizing AD and MCI brains (Liu et al., 2015a). A diversity of biomarkers extracted from the PET and/or MRI data have been proposed in previous studies. Liu et al. recently gave a comprehensive review of these biomarkers (Liu et al., 2015b). The most well-known MRI biomarkers include the regional gray matter volume (GMV), e.g., hippocampus and ventricles (Klöppel et al., 2008; Heckemann et al., 2011), cortical thickness (Fischl and Dale, 2000; Dickerson et al., 2009; Frisoni et al., 2010), local gyrification index (Schaer et al., 2008), curvedness and shape index (Awate et al., 2010; Cash et al., 2012). PET-derived biomarkers are generally pertaining to the radioactive tracers. Amyloid-binding compounds, i.e., ^18^F-BAY94-9172, ^11^C-SB-13, ^11^C-BF-227, ^18^F-AV-45, and ^11^*C-Pittsburgh compound B* (^11^C-PiB), have been used for imaging amyloid plaques in AD (Carpenter et al., 2009; Perrin et al., 2009; Thompson et al., 2009; Ni et al., 2013), whereas *2-[*^18^*F]fluoro-2-deoxy-D-glucose* (FDG) has mainly been used to depict glucose metabolism (Minoshima et al., 1995; Cai et al., 2010). Various static and kinetic biomarkers can be extracted from the PET data, i.e., the standard uptake value (SUV) (Clark et al., 2012; Landau et al., 2013), cerebral metabolic rate of glucose consumption (CMRGlc) (Sokoloff et al., 1977; Cai et al., 2010), mean index (Batty et al., 2008), z-scores (Minoshima et al., 1995), hypo-metabolic convergence index (HCI) / amyloid convergence index (ACI) (Chen et al., 2011), tissue time activity curve (TTAC) (Cai et al., 2000). In our previous studies, we proposed the convexity ratio and solidity ratio (Liu et al., 2013a) to detect the brain atrophy with MRI and the Difference-of-Gaussian (DoG) features (Cai et al., 2014) to detect the hypo-metabolism with FDG-PET, respectively. All of these biomarkers have been proved to have great potentials of differentiating the AD and MCI patients from normal controls (NC), and they also have demonstrated different strengths in characterize the disease pathology, e.g., PET views, such as SUV and CMRGlc in FDG-PET, are effective in detecting the functional anomalies in the brain, whereas MRI views, such as GMV and cortical thickness, are more sensitive to the brain morphological changes (Fan et al., 2008; Desikan et al., 2009; Risacher et al., 2009).

Researchers have carried out many studies on fusing these multi-view features. As pointed out by Atrey et al. (2010) and Zhang et al. (2011), current multi-view fusion methods could be roughly categorized into two groups, i.e., feature fusion and decision fusion. The feature fusion methods create a new feature space for the multi-view features and subsequently train a single model to classify the patients. Feature selection is a special feature fusion algorithm, that selects the most discriminant features based on certain selection criteria, such as *t*-test (Heckemann et al., 2011), Lasso (Zhu et al., 2014), or Elastic Net (EN) (Shen et al., 2011). The advanced feature fusion methods include multi-view spectral embedding, which embed the multi-view feature spaces into a unified space based on manifold learning (Park, 2012; Liu et al., 2013c; Che et al., 2014), the multi-kernel support vector machine (MK-SVM) that combines the feature spaces with kernel tricks (Hinrichs et al., 2009, 2011; Zhang et al., 2011), and deep learning methods that extract highly abstract features with a multi-layered neural network (Liu et al., 2014b, 2015c). The decision fusion methods train different models for different views, and subsequently aggregate the predictions of the all classifiers to make the final decision. Decision fusion, as compared to feature fusion, requires repeatedly training the classifiers and tuning their weighting parameters. In our recent study (Liu et al., 2013b), we proposed the Multifold Bayesian Kernelization (MBK) method to synthesize the multi-view biomarkers. MBK could construct a set of non-linear kernels to obtain the classification probabilities for individual views, and then infer their weighting parameters by minimizing the diagnostic errors and kernelization errors based on a Bayesian framework.

The aforementioned studies show that multi-view biomarkers could achieve better performance than single biomarkers, and imply that the multi-view biomarkers could create the synergy in the classification of AD and MCI (Hinrichs et al., 2011; Zhang et al., 2011; Singh et al., 2012; Liu et al., 2013b, 2014a; Jacobs et al., 2015). However, researchers do not yet understand the cause of such synergy, and there is a lack of the methods for quantitatively analyzing the synergy between individual biomarkers. Therefore, this study differs from the other multi-view studies in that our interest is to investigate the synergy between the multi-view biomarkers instead of solely improving the staging performance.

We propose a cross-view pattern analysis framework to investigate the synergy between the multi-view biomarkers. With this framework, we found that the biomarkers derived from MRI and PET could be separated into four clusters, each having a unique strength in detecting certain pathological changes in AD and MCI. We evaluated these biomarkers in a task of classifying the AD, MCI, and NC subjects obtained from the ADNI baseline cohort, and found the inter-cluster combination could always achieve the best performance compared to the intra-cluster combination. This study does not require the ethical approval since it is purely based on the analysis of the medical imaging data with no involvement of the patients, and the permission has been obtained to use the ADNI datasets.

The reminder of this paper is organized as follows. In Section 2, we first described the ADNI datasets, the pre-processing steps and the multi-modal features used in this study, and then elaborated the single-view and cross-view pattern analysis methods as well as the classification and evaluation methods. The pattern analysis and classification results were shown in Section 3, followed by the discussion on our findings in Section 4. Finally we concluded in Section 5.

## 2. Materials and methods

**Figure 1:**
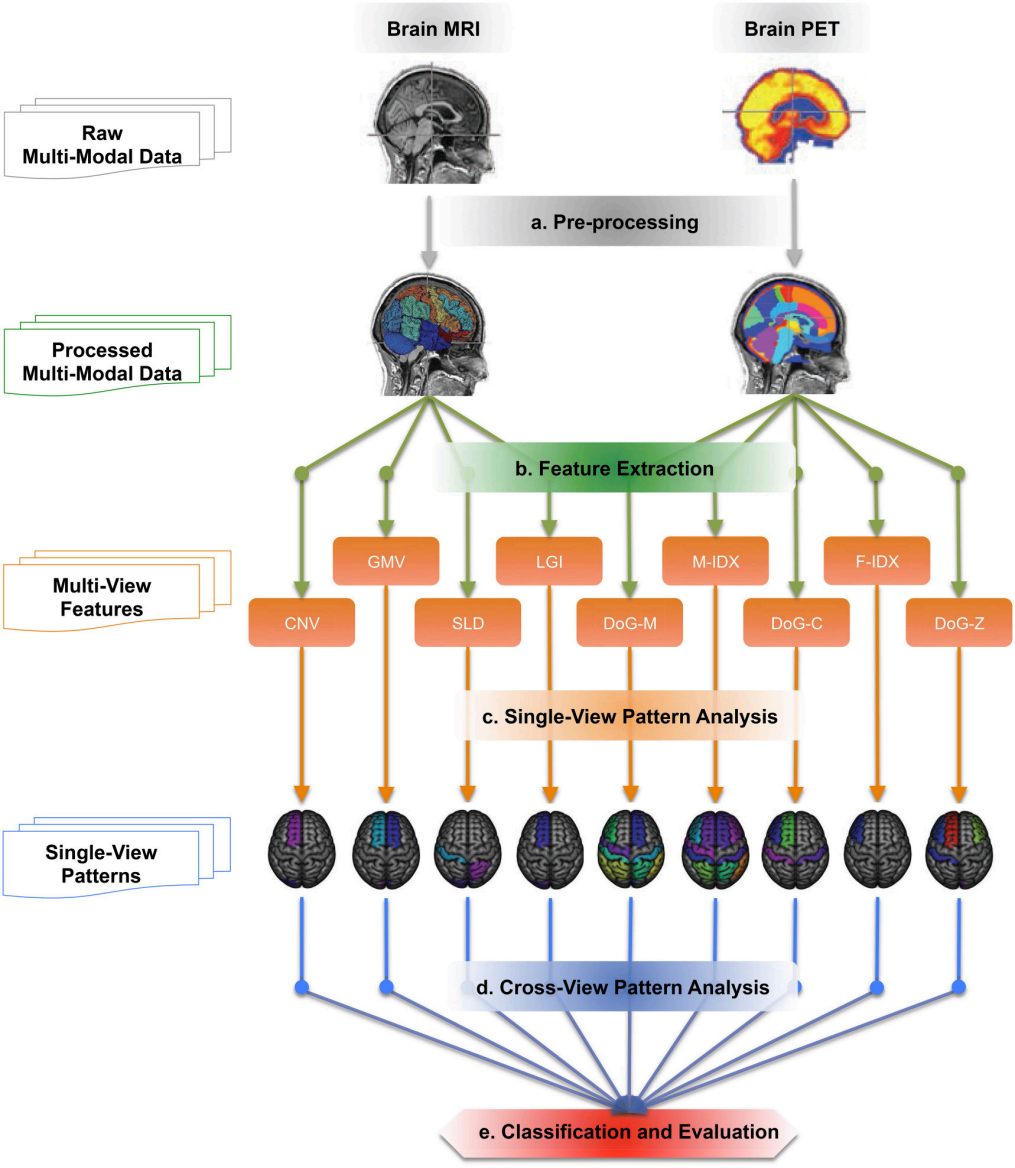
**The work-flow of the cross-view pattern analysis**. It is a five-step pipeline, which takes brain T1-MRI and FDG-PET images as inputs and generates the classification results as the outputs.

Figure 1 illustrates the work-flow of our analysis. We first acquired the raw MRI and PET datasets from the ADNI baseline cohort, then registered the brain volumes to a template and segmented them into a set of 3D regions of interest (ROI). Totally nine views of biomarkers were extracted from each ROI. Single-view and cross-view pattern analyses were carried out on these views based on their pathology patterns in terms of the brain atrophy and hypo-metabolism. Finally, we evaluated the single-view biomarkers and their combinations in the classification of AD, MCI, and normal control (NC) subjects using the MK-SVM algorithm.

### 2.1. Datasets

Data used in the preparation of this article were obtained from the Alzheimers Disease Neuroimaging Initiative (ADNI) database (adni.loni.usc.edu). The ADNI was launched in 2003 as a public-private partnership, led by Principal Investigator Michael W. Weiner, MD. The primary goal of ADNI has been to test whether serial magnetic resonance imaging (MRI), positron emission tomography (PET), other biological markers, and clinical and neuropsychological assessment can be combined to measure the progression of mild cognitive impairment (MCI) and early Alzheimer's disease (AD).

The ADNI datasets consist of a variety of imaging and non-imaging biomarkers, such as MRI, PET, cerebrospinal fluid (CSF) measures, genetic biomarkers, and clinical assessments. Our focus of this study was to investigate the neuroimaging biomarkers, therefore, we selected all of 369 subjects who had both the T1-weighted MRI volume scanned on a 1.5 Tesla MR scanner and the FDG-PET volume from the ADNI baseline cohort. After pre-processing, we visually checked the images and excluded those with intolerable distortions, that resulted in a downsized database of *N*(*N* = 331) subjects. These patients were divided into three groups according to their baseline diagnoses, including 85 AD, 169 MCI, and 77 NC subjects.

### 2.2. Pre-processing

In Step (a), as indicated in Figure 1, we pre-processed all these 3D MRI and PET volumes using the following protocols. We retrieved the MRI and PET volumes from ADNI database (Jack et al., 2008; Jagust et al., 2010). The PET data have a common isotropic voxel size of 1.5 *mm*^3^ and a full width at half maximum resolution of 8 mm. We then removed the non-brain tissue from MRI images using FSL BET (Smith, 2002). To enable the subsequent joint analysis of PET and MRI, i.e., feature extraction and pattern analysis, we then linearly registered the PET image to the MRI image of the same subject using FSL FLIRT (Jenkinson et al., 2002).

ROI-based features, as compared to voxel-based features, had lower dimensions and would avoid the curse-of-dimensionality. In addition, brain ROI features, such as hippocampal and ventricular volumes, have shown promising potential in characterizing AD and MCI. Therefore, we chose to use ROI-based features instead of voxel-based features in this analysis. The MRI data in ADNI baseline cohort have been labeled with *K*(*K* = 83) brain ROIs through the multi-atlas propagation with enhanced registration (MAPER) approach (Heckemann et al., 2010, 2011). These MAPER-generated labelmaps were then used to extract the ROI features in the next step. A complete list of these ROIs can be found in the previous papers (Heckemann et al., 2011; Liu et al., 2014a).

### 2.3. Feature extraction

As shown in Figure 1 - Step(b), totally *M*(*M* = 9) views of biomarkers were investigated in this study, including four biomarkers extracted from the T1-weighted MRI data: Gray Matter Volume (GMV), Local Gyrification Index (LGI), Convexity Ratio (CNV), and Solidity Ratio (SLD); and five biomarkers extracted from FDG-PET data: Mean Index (M-IDX), Fuzzy Index (F-IDX) and three Difference-Of-Gaussian features (DoG-M, DoG-C, DoG-Z). Since the features were all ROI-based, each feature element had two attributes, the location in the brain and the feature value. These two attributes together formed a signature neurodegeneration pattern of each view.

#### 2.3.1. Gray matter volume

GMV, is the most commonly used MRI biomarker in AD characterization in laboratories, since the GMV is closely related to the cortical neuronal loss as well as synaptic loss due to the disease (Carison et al., 2008). In this study, we extracted the GMV features from all *K* ROIs except for the ventricles, central structures, cerebellum and brainstem (whole volumes were used for these ROIs). We further normalized the GMV features by the intracranial volume as measured on the same source image to eliminate the impact of linear scaling in segmentation.

#### 2.3.2. Local gyrification index

LGI, is a metric that quantifies the ratio of the cortex buried within the sulcal folds to the outer visible cortex (Schaer et al., 2008). A normal healthy cortex with extensive folding usually has a larger LGI, whereas a degenerative cortex with limited folding has small LGI. The LGI features are usually computed in circular 3D ROIs in each hemisphere. In order to match the other views of features in this study, we computed the LGI features in the *K* pre-defined ROIs instead, i.e., the intersection of the 3D circular ROIs and pial surface were replaced by the outer surface of the pre-defined ROIs. For the non-cortical regions, the surface areas were used as the LGI features.

#### 2.3.3. Convexity ratio

CNV, also aims to capture the cortical folding features. CNV differs from LGI in that it is not limited to the cortex surfaces. It is defined as the area of the convex hull surface divided by that of the ROI surface (Liu et al., 2013a). Similar to LGI, a normal healthy brain usually has a larger CNV, and a degenerative brain has low CNV.

#### 2.3.4. Solidity ratio

SLD, quantifies the fullness of the ROI in the convex hull. It is defined as the ratio of volume of the ROI to that of the convex hull. SLD describes the extend to which the shape is convex or concave. Compared to the normal healthy brains, the degenerative brains with atrophy usually have a shrinking shape, which leads to a lower SLD value. SLD and CNV are usually used together to enhance the GMV features due to large inter-subject brain volume variations (Liu et al., 2013a).

#### 2.3.5. Mean index

M-IDX, is defined as the mean activity levels of the ROIs (Batty et al., 2008). It is a simple and effective feature in capturing the brain metabolism activity levels and has been widely used in AD and MCI characterization. In particular, M-IDX is very sensitive to the brain hypo-metabolism and has better performance in early detection of MCI than many complex feature descriptors, such as 3D Gabor Filters, Gray Level Co-occurrence Matrix, and Discrete Curvelet (Liu et al., 2014a). To eliminate the intensity variations during acquisition or parameter estimation, we further normalized the M-IDX features with the average cerebellum metabolism rate.

#### 2.3.6. Fuzzy index

F-IDX, evaluates the consistency of the metabolism activity levels, or the fuzziness, of the ROIs. It is defined as the standard deviation divided by the mean value of the ROI voxels. F-IDX is particularly useful for characterizing the ROIs that are partially hypo-metabolic. The voxels in these ROIs have less consistent activity levels, thus lead to higher F-IDX. On the contrary, the normal ROIs are expected to have more consistent activity levels and smaller F-IDX values.

#### 2.3.7. Difference-of-Gaussian mean

DoG-M, quantifies the degeneration levels of the hypo-metabolic regions (lesions) at different spatial scales estimated by the Difference-of-Gaussian (DoG) descriptor. It is defined as the mean metabolism rate of the lesion area within the segmented ROI (Cai et al., 2014). Different from M-IDX, DoG-M considers the activity level of the lesions only. The mean metabolism rate of all lesion areas across the brain is first computed, and further normalized by the mean metabolism rate of the cerebellum to remove the bias of global intensity variation. It is originally called the lesion mean index. To avoid the ambiguity with M-IDX, we referred to it as DoG-Mean (DoG-M) in the rest of this paper.

#### 2.3.8. Difference-of-Gaussian contrast

DoG-C, quantifies the contrast between the lesions and non-lesion parts. Since there are large variations of the metabolism rates in different ROIs, DoG-C offsets this effect by focusing on the contrast instead of the actual activity level of the ROI. It is originally called the lesion contrast index and defined as the ratio of the mean metabolism rate of the lesions to that of the non-lesion parts and further corrected using the variances of both parts in the ROIs, where the lesions are also approximated by the DoG descriptor.

#### 2.3.9. Difference-of-Gaussian Z-score

DoG-Z, similar to the conventional Z-score (Minoshima et al., 1995), quantifies the proportion of the abnormal voxels in the ROIs. However, conventional Z-score requires voxel-wise registration which will involve registration error, instead we used DoG operator to estimate the hypo-metabolism lesions in this study. DoG-Z is a good indicator to approximate the progress of the disease. Late-stage patients usually have higher DoG-Z values than the early stage patients.

### 2.4. Single-view pattern analysis

In single-view pattern analysis, as shown in Figure 1 - Step(c), we analyzed the pathology patterns of the nine individual views extracted from the imaging data.

For each view, we performed ANOVA on the three disorder groups, AD, MCI, and NC, against the null hypothesis that all groups were simply random samples of the same population. Given a view, *P*, the *p*-values of ANOVA, *P* = {*P*(1), *P*(2), …, *P*(*K*)}, showed the discriminating power of the ROIs in this view. To make it comparable to other views' patterns, we transformed the *p*-values to non-negative valued weights, which were positively correlated to the ROI discriminating power, as Equation (1):

(1) $$P\prime (i)=\exp(-\frac{P{\left(i\right)}^{2}}{2\sigma^{2}})$$

where σ is the bandwidth parameter which controls how quickly *P*′(*i*) falls off with the *P*(*i*). If *P*(*i*) is small, then *P*′(*i*) is close to 1; and if *P*′(*i*) is greater than σ, then *P*′(*i*) will plummet to 0. In this study, we set the bandwidth σ as 0.05.

In order to quantify the differences between the patterns in the following analysis, we further normalized the *P*′ as Equation (2):

(2) $$P\prime \prime (i)=\frac{P\prime (i)}{\sum_{K}P\prime \left(j\right)}$$

The normalized weights, *P*″(1), *P*″(2), …, *P*″(*K*), together formed a distinct pathology pattern of the view.

There are three types of ROIs in terms of their consistency across different views. The first type of ROIs is the disease-spared ROIs, which are not affected by the disease and have low discriminating power across most of the views, e.g., cerebellum is believed to be spared by AD and always used to calibrate the PET metabolism rates. The second type of ROIs is the disease-affected ROIs. Hippocampus, for instance, has been widely used as an effective biomarker for characterizing AD and MCI. The third type of ROIs is the view-specific ROIs, which have varying *p*-values across different views. These ROIs show the different effects of the disease on the brain, and potentially lead to the synergy or interference between different views.

### 2.5. Cross-view pattern analysis

Figure 1 - Step(d) shows the cross-view pattern analysis. The goal of this step was to compare the patterns and quantitatively analyze the variability among them.

We first paired up these *M* views, which lead to *M* × (*M* − 1)/2 pairs of views. In this study, there were 9 single views and 36 pairs. We then quantitatively analyzed each pair based on their patterns. Assuming *P*″ and *Q*″ represent the patterns of two views, we computed their affinity, *A*(*P, Q*), as Equation (3):

(3) $$A(P,Q)=\exp\left(\text{​​}-\frac{1}{2\pi }(\overset{D_{KL}(P||Q)}{\overset{︷}{\sum_{K}P\prime \prime (i)\log\frac{P\prime \prime (i)}{Q\prime \prime (i)}}}\text{​}+\text{​}\overset{D_{KL}(Q||P)}{\overset{︷}{\sum_{K}Q\prime \prime (i)\log\frac{Q\prime \prime (i)}{P\prime \prime (i)}}})\right)$$

where *D*_*KL*_(*P*||*Q*) is the Kullback-Leibler (KL) divergence of *Q* from *P*, and *D*_*KL*_(*Q*||*P*) is the KL divergence of *P* from *Q*. *A*(*P, Q*) = 0 if *P*″ = *Q*″. Note that KL divergence is non-symmetric measure of difference between *P* and *Q*, and cannot be used as a distance metric as it does not satisfy the symmetry condition. Therefore, we actually measured the affinity between two views based on their mutual divergence.

The affinity value of all pairs formed the affinity matrix *A*. To see how the views were related to each other, we further computed the clustering of them based on the symmetric normalized Laplacian matrix (*L*) of *A* (Ng et al., 2002), as Equation (4):

(4) $$L=I-D^{-1/2}AD^{-1/2}$$

where *I* is the unit matrix, *D* is defined as the diagonal matrix whose (*i, i*)-element was the sum of *A*'s *i*th row. If we consider the patterns to be the points in a *K*-dimensional space, then the top-*k* eigenvectors of *L* could be stacked in columns to form a new *k*-dimensional space for the patterns, therefore it allowed us to observe the embedding of the views in a low dimensional space. In this study, we set *k* to 2 and displayed the views as points in a 2-dimensional space.

### 2.6. Classification and evaluation

The last step of our work-flow was to evaluate the performance of these 9 single views and 36 pairs of views in the task of staging of the disease progression, i.e., classifying the AD, MCI and NC subjects, as illustrated in Figure 1 - Step(e). The goal of this step is to see how the single-view biomarkers interact with each other and find out what biomarkers have more effective synergy than others.

Since the datasets used in this study were highly skewed that MCI subjects accounted for a large percentage (over 50%) of the entire population, we designed three classifiers instead of one classifier in order to reduce the data bias onto classification and achieving more accurate staging. The first classifier was a binary SVM aiming to distinguish NC subjects from the AD and MCI patients. We kept the NC subjects predicted by the first classifier and sent other subjects to the second classifier. The second classifier was also a binary SVM, which classified the subjects into AD or non-AD patients. The predicted AD patients of the second classifier were retained and the rest of the patients were sent to the third classifier. The third classifier was a multi-class SVM, which classified AD, MCI, and NC subjects in one setting. The Radial-Basis-Function SVM (RBF-SVM) was used for the single views, whereas the Multi-Kernel SVM (MK-SVM) was used for the combinations of the views. Both the RBF-SVM and MK-SVM were implemented using LIBSVM library (Chang and Lin, 2011).

The 5-fold cross-validation paradigm was adopted in performance evaluation. Specifically, we divided the datasets into 5 equal-sized subsets, and each subset was used for testing in turn while other subsets were used for training the model. While training, the three classifiers were trained together and the hyper-parameters were optimized using the random search optimization algorithm (Bergstra and Bengio, 2012). Totally six performance metrics were used in this study, including three precision metrics for AD, MCI, and NC respectively, and the overall accuracy, specificity and sensitivity. Note that when computing the specificity and sensitivity, NC was considered as the negative class, and both MCI and AD were considered as the positive class. The corresponding standard deviations from cross-validation were also reported with the performance metrics.

## 3. Results

### 3.1. Single-view pattern analysis result

**Figure 2:**
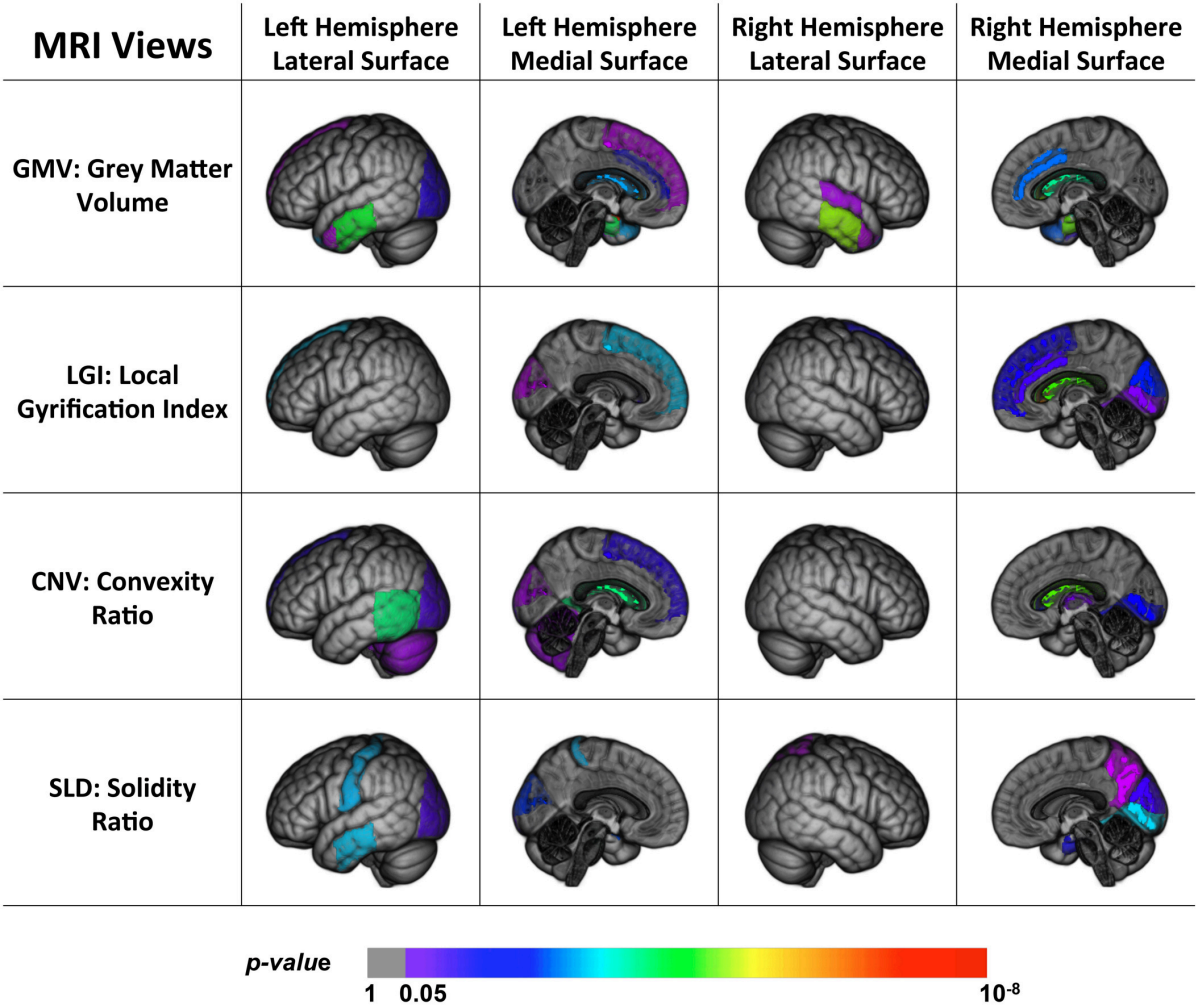
**Back projection of the normalized weights of the ROIs for four MRI views onto the ICBM_152 template using 3D Slicer (Fedorov et al., 2012)**.

Figure 2 shows the back-projection of the MRI single-view patterns onto the ICBM_152 brain template (Mazziotta et al., 2001), which is also labeled using the MAPER approach. The color bar indicates the *p*-values of the ROIs in each view. Note that the ventricles and corpus callosum are not displayed here. Based on these patterns, we found that a large proportion of the brain was spared by the disease, such as the insula, brain stem, corpus callosum, and parts of the frontal lobe, parietal lobe and subcortical regions. The disease-affected regions include the repeatedly reported ventricles, middle and inferior temporal lobe and limbic gyrus. We also observed a strong agreement across most views on parts of the occipital lobe (lateral part, lingual, and cuneus) and frontal lobe (superior part), which were less investigated in previous studies. GMV further detected the hippocampus, parahippocampal and ambient gyrus, and amygdala. CNV detected two particular ROIs, the cerebellum and the thalamus, although these two structures were usually considered spared by AD. SLD also has two signature ROIs in the parietal lobe, including the superior and post-central parts.

**Figure 3:**
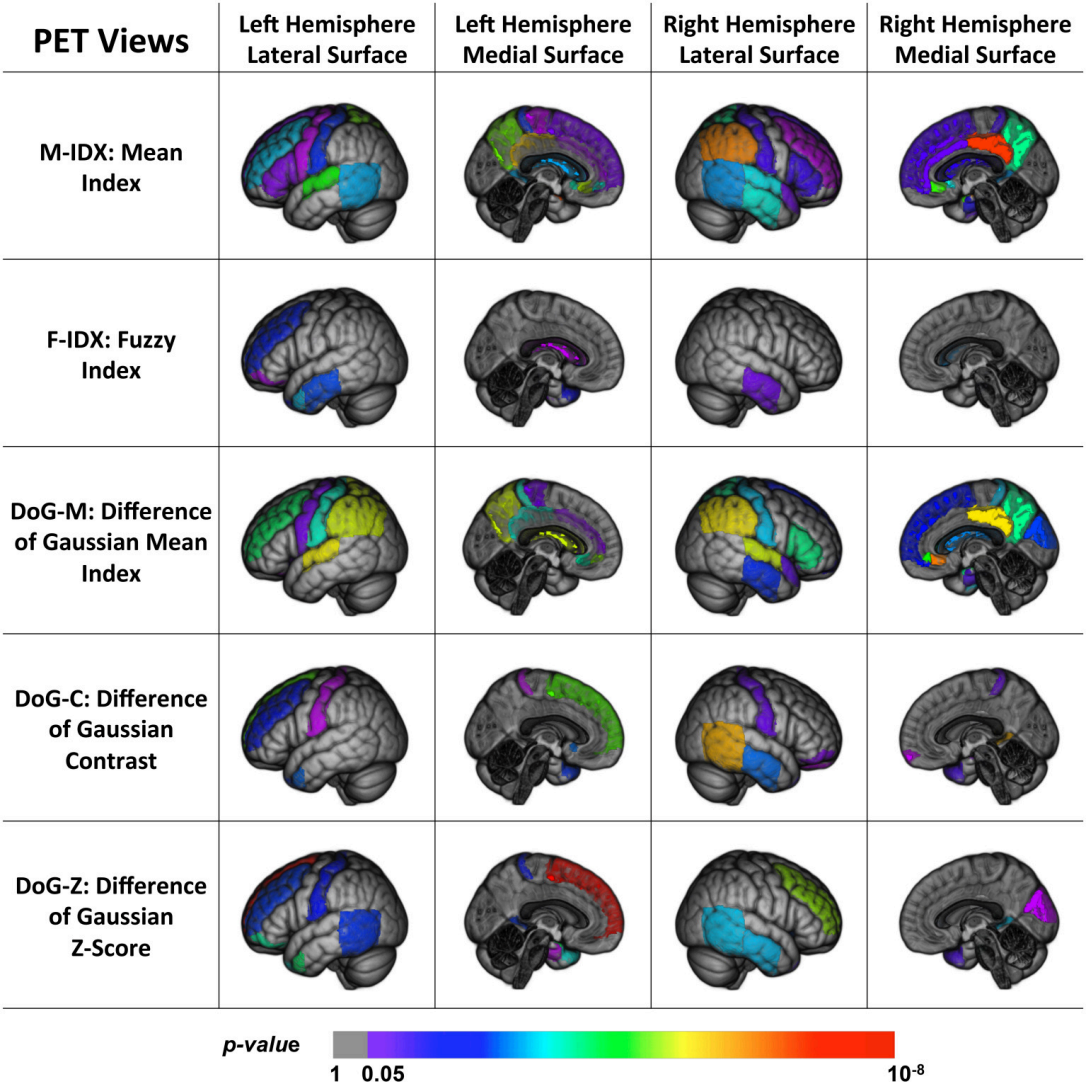
**Back projection of the normalized weights of the ROIs for five PET views onto the ICBM_152 template using 3D Slicer**.

Figure 3 shows the back-projection of the PET single-view patterns onto the ICBM_152 brain template. In addition to the temporal lobe and limbic gyrus that were detected by MRI views, the PET patterns also included more frontal (subgenual, orbital, inferior, middle, and superior parts) and parietal areas (post-central and superior parts). These regions are believed affected at the later course of AD and MCI, after the hippocampus, entorhinal cortex, temporal regions and posterior cingulate (Fan et al., 2008). This indicated that frontal and parietal lobe were essential in staging AD and MCI, and we may more effectively detect functional changes rather than structural changes in these regions. Compared to MRI views, they were less sensitive to pathological changes in the occipital lobe, where only the cuneus was detected by the DoG-M and DoG-Z. The patterns of M-IDX and DoG-M were larger than the other views, both covering the inferiolateral parietal area.

To summarize, we found that parts of the brain were disease-spared regions verified by both PET and MRI views. MRI views were capable of capturing the brain structural changes on temporal lobe, limbic gyrus, the ventricles, and part of the occipital lobe, which were usually shaped in the late course of the disease. The PET views, on the other hand, reflected the metabolic activities of the brain and were able to detect the early functional anomalies, therefore they tended to involve more ROIs in their patterns than the MRI views, especially in the frontal and parietal areas. In addition, some ROIs could only be detected by certain views, and led to distinct patterns. The differences of these patterns indicated that the disease had different effects on the brain and no single-view biomarkers were able to capture all the pathological changes.

### 3.2. Cross-view pattern analysis result

Table 1 shows the KL divergence (*D*_*KL*_(*Col*||*Row*)) of the row item (*Row*) from the column item (*Col*) for these nine views. PET views had a low mean KL divergence of 16.6, which was close to that of MRI views 17.8. However, the mean KL divergence of PET views from MRI views (*D*_*KL*_(*MRI*||*PET*) = 47.37) was markedly higher than that of MRI views from PET views (*D*_*KL*_(*PET*||*MRI*) = 20.87). These results indicated that the views in the same modality usually look more similar than those in different modality. A typical example to show inter-modal and intra-modal differences was the GMV, which had limited divergence from other MRI views (LGI:6.83; CNV:2.31; SLD:3.99), but large divergence from PET views (M-IDX:48.09; F-IDX:31.13; DoG-M:49.95; DoG-C:39.28; DoG-Z:33.53). In addition, the MRI views always gain more information from the PET views than otherwise, e.g., *D*_*KL*_(*M*-*IDX*||*CNV*) = 51.80 is much greater than *D*_*KL*_(*CNV*||*M*-*IDX*) = 4.01; *D*_*KL*_(*DoG*-*M*||*LGI*) = 50.15 is also greater compared to *D*_*KL*_(*LGI*||*DoG*-*M*) = 9.21. The only exception was the pair of CNV and DoG-Z, both having high divergence from each other. As for the individual views, the divergence had a very wide range from the minimum *D*_*KL*_(*M*-*IDX*||*DoG*-*M*) = 0.35 to the maximum *D*_*KL*_(*SLD*||*DoG*-*C*) = 61.47.

**Table 1:** **The cross-view Kullback-Leibler divergence between different views**.

***D_KL_(Col||Row)***		**GMV**	**LGI**	**CNV**	**SLD**	**M-IDX**	**F-IDX**	**DoG-M**	**DoG-C**	**DoG-Z**
MRI	GMV	0	32.40	33.73	25.00	5.19	23.22	12.59	28.42	16.81
	LGI	6.83	0	16.26	28.30	3.39	21.67	9.21	28.15	22.30
	CNV	2.31	20.39	0	38.71	4.01	19.39	28.44	45.88	46.15
	SLD	3.99	32.88	25.40	0	5.37	39.22	11.65	31.84	14.30
PET	M-IDX	48.09	48.93	51.80	49.83	0	39.06	6.03	45.31	34.59
	F-IDX	31.13	43.04	50.26	55.67	13.00	0	18.87	18.04	9.85
	DoG-M	49.95	50.15	56.92	47.56	0.35	42.70	0	55.06	35.05
	DoG-C	39.28	52.29	40.63	61.47	6.92	27.94	18.63	0	13.31
	DoG-Z	33.53	43.80	45.11	47.93	9.19	27.52	19.32	4.10	0

**Figure 4:**
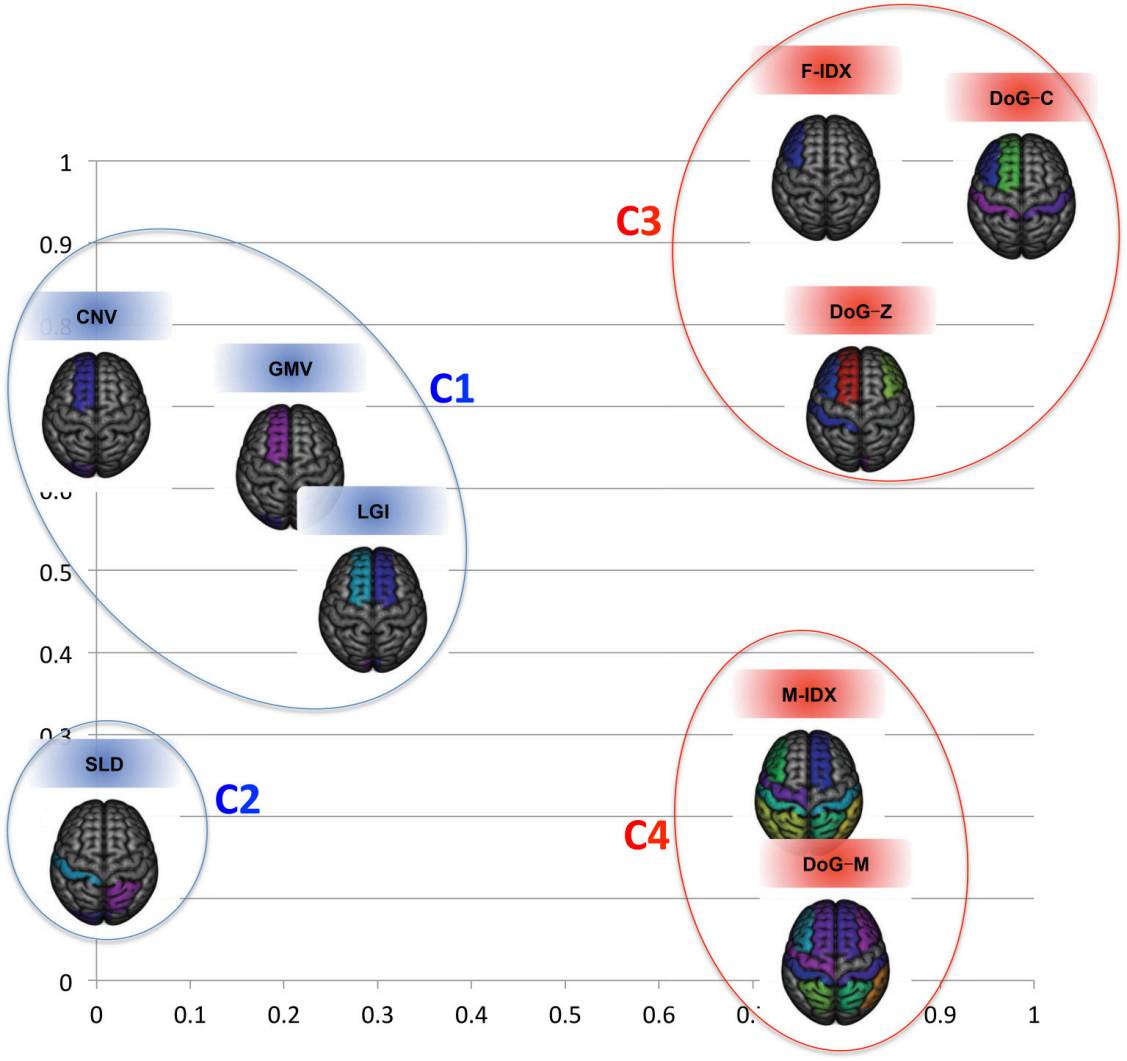
**The clustering results for the nine views in the 2D space**. The structural and functional features are substantially separated when considering the first two eigenmodes.

To see how individual views related to each other, Figure 4 displays their clustering results in a 2D space using the cross-view pattern analysis method described in Section 2.5. The blue color indicates the MRI views, the red color indicates the PET views, and the distance between two views in this coordinate system is proportional to their mutual divergence. We noticed that the MRI and PET views were clearly separated. More importantly, these views also formed clusters within the same modality. There were two clusters for the MRI views and two clusters for the PET views. The first sub-cluster (C1) for MRI included CNV, GMV, and LGI. All of these three features had strong correlation with the brain cortical atrophy, such as the loss of cortical neurons, the changes of cortical foldings. The second cluster (C2) for MRI had one isolated view only, SLD. Different from other MRI views, SLD focused on the shape changes of the brain caused by the disease. The third cluster (C3) contained three PET views, F-IDX, DoG-C, and DoG-Z. These views were effective in evaluating the consistency of the activity levels within a ROI, particularly when the ROI was partially hypo-metabolic. The M-IDX and DoG-M formed the fourth cluster (C4). These two views both were sensitive to the metabolic activity changes of the brain, which were important in the early detection of the AD and MCI.

### 3.3. Single-view classification performance

The classification performances of the individual views are summarized in Table 2. The best result of each performance metric is highlighted in bold-face. In general, PET views tended to have better performance than MRI views, especially on NC precision, MCI precision and the overall specificity. The only exception was DoG-Z, which had lower NC precision, MCI precision, accuracy and sensitivity compared to the MRI views. In addition, the sensitivity was always higher than the specificity across all the views, with an average difference of 30.44%. This was because we considered both AD and MCI as the positive class when computing the sensitivity and specificity. We argued that sensitivity was more important than specificity in this classification task, because the strong ability to detect the positive class (AD and MCI) would avoid treating the patients as normal subjects.

**Table 2:** **The classification performance of single-view biomarkers**.

**Method**		**NC**	**MCI**	**AD**	**Accuracy**	**Specificity**	**Sensitivity**
MRI	GMV	43.08±11.48	60.23±8.30	**67.64 ± 22.93**	55.58±5.49	48.33±10.26	78.76±11.68
	LGI	31.27±6.38	52.34±12.98	44.04±12.75	44.42±9.78	35.17±7.87	76.35±4.37
	CNV	43.91±4.28	57.57±4.25	50.63±12.94	52.27±5.92	47.58±17.96	81.45±7.38
	SLD	39.86±8.60	55.64±3.56	47.09±5.66	49.24±1.99	48.17±16.29	78.37±4.50
PET	M-IDX	45.46±7.96	60.88±3.57	62.19±13.46	56.20±3.06	51.83±10.61	81.11±4.27
	F-IDX	**53.30 ± 10.55**	**64.06 ± 3.69**	49.47±6.32	**56.49 ± 3.89**	**63.67 ± 8.52**	81.90±7.75
	DoG-M	49.02±14.52	63.20±7.12	55.39±11.66	56.19±4.68	50.58±4.20	**82.27 ± 7.99**
	DoG-C	44.84±8.92	57.56±5.33	55.83±7.00	52.88±4.56	52.33±19.49	79.47±10.27
	DoG-Z	33.84±2.41	51.95±3.62	51.91±8.26	46.54±2.89	42.92±8.83	74.82±3.06

The bold value is the highest value in each column.

It was very clear that no single view could win all. F-IDX was the best view with the highest NC precision (53.30%), MCI precision (64.06%), overall accuracy (56.49%), and specificity (63.67%). GMV achieved the highest AD precision (67.64%), and DoG-M had the highest sensitivity (82.27%). One interesting discovery about the top three views (F-IDX, GMV, and DoG-M) was that they were from three distinct clusters (C3, C1, and C4), as described in Section 3.2. This fact implied that different views had different strengths in classification, and such strengths might be related to the clusters they belonged to.

### 3.4. Cross-view classification performance

Table 3 shows the classification performance of 36 pairs of biomarkers in the same classification task. The best result of each performance metric is highlighted in bold-face. We separated the pairs into three groups according to their modalities, including six intra-MRI pairs, 10 intra-PET pairs, and 20 inter-PET&MRI pairs.

**Table 3:** **The classification performance of the 36 combinations of the multimodal neuroimaging biomarkers**.

**Method**		**NC**	**MCI**	**AD**	**Accuracy**	**Specificity**	**Sensitivity**
Intra-MRI	GMV / LGI	53.90±9.39	63.83±1.85	72.49±18.80	64.28±3.94	32.58±13.38	91.31±4.43
	GMV / CNV	60.83±22.54	66.81±5.87	71.67±7.45	63.43±3.74	54.75±25.44	84.60±11.41
	GMV / SLD	53.18±11.18	67.49±3.87	72.73±13.31	63.13±5.61	52.17±18.78	83.07±14.32
	LGI / CNV	50.71±8.17	60.82±3.69	69.00±18.17	58.01±4.84	54.58±18.37	82.25±9.31
	LGI / SLD	44.17±3.37	64.01±3.74	57.64±7.93	56.81±2.29	45.58±21.85	82.29±9.49
	CNV / SLD	43.09±5.66	64.40±8.22	62.11±9.95	55.59±3.53	64.83±14.84	74.42±3.56
Intra-PET	M-IDX / F-IDX	55.00±2.65	68.22±4.82	64.26±9.03	62.55±2.03	68.58±17.62	82.65±5.75
	M-IDX / DoG-M	54.64±6.08	65.06±2.66	69.42±16.09	62.85±3.66	50.33±16.17	87.39±4.13
	M-IDX / DoG-C	59.48±14.50	64.54±1.69	67.94±8.80	62.85±3.82	55.67±10.50	86.60±8.30
	M-IDX / DoG-Z	51.75±4.03	62.15±3.50	68.55±13.25	61.04±3.73	47.67±18.92	86.21±5.75
	F-IDX / DoG-M	65.70±13.38	68.84±5.29	73.07±13.09	**67.37 ± 2.10**	51.92±18.19	91.36±5.63
	F-IDX / DoG-C	56.99±8.28	63.19±3.47	65.93±7.93	60.72±1.98	67.67±12.66	83.47±7.54
	F-IDX / DoG-Z	55.11±12.68	64.15±1.32	68.35±19.06	60.71±2.57	59.75±5.22	83.86±7.77
	DoG-M / DoG-C	57.33±9.39	63.79±4.67	65.78±12.46	61.93±6.74	53.25±18.99	86.60±9.18
	DoG-M / DoG-Z	52.79±7.67	64.53±7.03	68.27±12.69	61.94±4.38	52.83±22.03	85.01±6.98
	DoG-C / DoG-Z	46.16±7.88	62.98±5.35	68.66±18.96	58.32±4.09	51.08±21.75	82.31±7.54
Inter-MRI & PET	GMV / M-IDX	51.04±4.05	67.69±5.26	76.08±17.25	63.75±5.74	57.00±18.27	83.07±6.31
	GMV / F-IDX	63.86±10.71	64.47±3.04	**80.56 ± 14.72**	65.86±4.85	54.33±22.96	88.98±8.72
	GMV / DoG-M	55.28±9.47	**70.89 ± 7.38**	75.78±22.48	64.94±4.84	57.00±20.01	85.00±8.21
	GMV / DoG-C	58.06±9.15	64.26±3.89	78.94±15.07	65.26±4.11	39.33±20.02	90.96±5.28
	GMV / DoG-Z	50.57±13.28	61.66±6.45	70.44±15.25	60.11±4.22	45.67±22.53	85.84±9.83
	LGI / M-IDX	62.90±21.27	63.88±4.15	69.47±16.10	61.65±3.23	45.25±17.98	88.56±6.90
	LGI / F-IDX	58.09±7.42	62.72±2.94	61.83±11.79	60.72±2.50	61.00±10.50	86.22±5.00
	LGI / DoG-M	57.89±13.26	63.50±3.80	67.33±20.05	60.73±3.43	45.00±20.74	87.77±8.54
	LGI / DoG-C	49.01±12.59	62.54±10.77	59.33±8.31	56.82±6.00	53.75±25.20	82.71±8.77
	LGI / DoG-Z	49.40±16.61	60.14±4.61	70.41±20.40	56.49±6.55	47.42±23.58	79.99±15.01
	CNV / M-IDX	64.92±21.45	63.25±5.57	65.00±10.63	61.04±3.23	43.92±23.57	88.60±10.49
	CNV / F-IDX	60.04±9.77	63.82±3.23	73.39±17.07	63.42±4.75	53.25±14.31	88.19±6.20
	CNV / DoG-M	**65.94 ± 7.53**	63.01±5.48	71.38±5.27	64.97±5.29	40.08±11.82	**93.69 ± 2.58**
	CNV / DoG-C	48.10±8.06	68.06±6.69	61.40±7.99	58.01±2.38	**69.92 ± 14.01**	75.13±11.62
	CNV / DoG-Z	51.43±5.45	60.89±3.47	68.11±19.51	58.31±4.43	46.08±26.15	85.35±12.28
	SLD / M-IDX	56.56±25.18	65.23±8.17	70.07±12.33	59.82±6.16	62.83±21.53	79.22±14.84
	SLD / F-IDX	54.64±7.30	65.28±3.77	61.88±10.26	60.41±3.90	68.75±11.87	81.91±7.21
	SLD / DoG-M	57.61±19.28	69.84±8.70	68.64±18.33	63.54±5.71	55.92±15.00	83.91±12.34
	SLD / DoG-C	49.25±4.79	63.48±7.01	74.13±9.40	58.62±2.52	66.67±23.22	78.38±9.75
	SLD / DoG-Z	46.68±8.64	56.65±4.21	62.01±10.75	54.67±6.15	49.50±18.92	81.53±11.94

The bold value is the highest value in each column.

Most of the biomarker pairs could outperform the single biomarkers with marked improvements. Similar to the single-view biomarkers, none of the pairs could be leading in all aspects. The pair of CNV and DoG-M achieved the best precision for NC at 65.94%, which was 12.64% higher than the best single-view performance. They also had the highest sensitivity of 93.69%, increased the best single-view sensitivity by 11.42%. The pair of GMV and F-IDX performed best on MCI classification with a precision of 70.89%, improved the best single-view MCI precision by 6.83%. GMV also had the best performance on AD classification when paired with F-IDX, and their AD precision was 80.56%, 12.92% higher than the best single-view AD precision. F-IDX had the best single-view accuracy of 56.49%, and it further improved the accuracy to 67.37% when combined with DoG-M. The highest specificity was 69.92%, achieved by CNV and DoG-C with an increase of 6.25% compared to the best single-view specificity.

We noticed that the inter-MRI&PET pairs usually gave better results than the intra-PET and intra-MRI pairs. As detailed above, the best results were always obtained from the inter-MRI&PET pairs, except for the overall accuracy, which was achieved by two PET views. However, when the views were separated into different clusters as in Figure 4, we found the two views in the best pairs were always from different clusters with no exception, i.e., C1 (CNV) and C4 (DoG-M) achieved best NC precision and overall sensitivity; C1 (GMV) and C4 (DoG-M) had best MCI precision; C1 (GMV) and C3 (F-IDX) led in AD precision; C3 (F-IDX) and C4 (DoG-M) attained highest overall accuracy; and finally C1 (CNV) and C3 (DoG-C) achieved best specificity. C2 (SLD) was the only cluster that made no contribution to any of the best results.

In summary, there were two clear tendencies based on the cross-view results. First, the biomarker pairs could achieve much better results than the single-view biomarkers. Second, the best performance was always achieved by the views from different clusters.

## 4. Discussions

The mutual divergence was an effective measure to quantize the variability of the biomarkers. In this study, we identified four clusters of the biomarkers based on their mutual divergence, and the best joint performance in classification was always achieved by the combination of views from different clusters. However, it was not clear whether mutual divergence could be used as a general performance predictor for any two biomarkers. Therefore, we further asked this question, what could we expect from the biomarkers when combining them in classification.

To answer this question, we first looked at the correlation between the joint performance of the biomarker pair and performance of individual biomarkers. We used the *E*_*joint*_, *E*_*high*_, and *E*_*low*_ to represent the joint performance, the higher performance and lower performance of the biomarkers. Table 4 shows the Pearson's Correlation Coefficients (ρ) and the corresponding *p*-values with a significant value of 0.05. It was very clear that the multi-view classification performance was strongly correlated to the performance of individual views, and largely affected by the view with higher performance.

**Table 4:** **Pearson's correlation coefficient of the performance and the divergence**.

***E*_*joint*_**	**NC**		**MCI**		**AD**		**Accuracy**		**Specificity**		**Sensitivity**	
	**ρ**	***p*-value**	**ρ**	***p*-value**	**ρ**	***p*-value**	**ρ**	***p*-value**	**ρ**	***p*-value**	**ρ**	***p*-value**
*E*_*high*_	0.43	3.1e-17	0.43	2.5e-17	0.62	6.6e-39	0.66	8.2e-47	0.51	2.3e-25	0.26	3.4e-07
*E*_*low*_	0.31	2.1e-09	0.35	1.6e-11	0.40	4.1e-15	0.45	4.7e-19	0.21	5.0e-05	0.13	1.5e-02
*D*_*mutual*_	0.00	1.0e+00	−0.03	5.6e-01	−0.07	1.6e-01	−0.10	4.8e-02	0.09	9.8e-02	−0.07	2.2e-01
*D*_*high*_	0.11	4.0e-02	0.02	6.5e-01	−0.03	5.4e-01	−0.01	9.0e-01	0.05	3.6e-01	−0.00	9.5e-01
*D*_*low*_	−0.12	2.7e-02	−0.08	1.3e-01	−0.09	8.1e-02	−0.17	9.8e-04	0.10	6.1e-02	−0.11	4.1e-02

We further examined the correlation between the joint performance and the mutual divergence *D*_*mutual*_, as well as the higher KL divergence *D*_*high*_ and lower KL divergence *D*_*low*_ between the two views. A large *D*_*high*_ and a large *D*_*low*_ mean that the two views have dramatically different patterns, such as DoG-Z and CNV. A large *D*_*high*_ and a small *D*_*low*_ mean one pattern covers the other, such as M-IDX and CNV. If the *D*_*high*_ and *D*_*low*_ are both small, then the patterns are very similar, such as DoG-M and M-IDX. As shown in Table 4, the mutual divergence *D*_*mutual*_ did not show a correlation with the joint performance *E*_*joint*_, except that it had a weak anticorrelation with the accuracy (ρ = −0.1, *p_value* = 0.048). The higher KL divergence *D*_*high*_ had a weak correlation with the NC precision (ρ = 0.11, *p_value* = 0.040), whereas the lower KL divergence *D*_*low*_, showed a decreasing linear relationship with the NC precision (ρ = −0.12, *p_value* = 0.027), accuracy (ρ = −0.17, *p_value*~ = 0.001), and sensitivity (ρ = 0.11, *p_value* = 0.041). In other words, an increase of *D*_*high*_ or a decrease of *D*_*low*_ might lead to better classification performance. Large divergence does not necessary lead to better performance, as the views might not only create synergy, but also cause the interference to each other.

Therefore, our answer to the above mentioned question is that the multi-view performance is primarily determined by the performance of individual views. If one view's pattern covers the other, they tend to perform better than those with highly different or similar patterns.

There are also limitations of the datasets and the classifiers of this study. The datasets used in this study consisted of 331 subjects, but these subjects were not evenly distributed in each group, i.e., MCI patients accounted for over 50% of the entire population. The large disparity of number of patients in individual groups might have an impact on the SVM classifier. To offset such impact, we designed a cascade of three classifiers instead of only one classifier to increase the chance of classifying NC and AD. However, there might be a high correlation between classifiers, which might result in redundancy and consequent reduced performance. Such design is rather *ad-hoc* and would not be necessary for the future datasets with evenly distributed patients in each group. In this study, we adopted a design of 3-class classification (AD / MCI / NC) with a focus on the staging of the disease. However, such design poses great challenges to interpret our detected patterns of ROIs, since we don't see which regions are significant for AD or MCI. In addition, MCI is essentially a heterogeneous group and a substantial number of MCI subjects had primary non-AD pathologies, such as vascular dementia (VD) and frontotemporal dementia (FTD), as suggested by a recent ADNI study (Nettiksimmons et al., 2014). Therefore, it will be particularly useful to further distinguish the MCI subjects, including stable MCI patients not converting to other pathologies (ncMCI), and MCI converters who convert to AD (cMCI) or other pathologies. Totally nine views of neuroimaging biomarkers were investigated in this study. All of the biomarkers were based on the same template with 83 pre-defined ROIs, thus their patterns can be compared to each other. However, the multi-modal biomarkers might not always be ROI-based, such as the voxel-based features and the non-imaging biomarkers. In addition, certain biomarkers were able to bring additional information than the ROI-based features. For instance, the popular connectome (Wang et al., 2014) derived from DTI could not only capture the features of the ROIs, but also quantize the correlation between them. Currently, our cross-view analysis framework could quantitatively analyze and predict the synergy between two biomarkers. However, it is still very challenging to predict the synergy of more than two biomarkers.

## 5. Conclusions and future work

In this study, we presented a cross-view pattern analysis framework to quantitatively analyze the synergy between the multi-modal biomarkers derived from T1-MRI and FDG-PET, and predict their performance in AD and MCI classification. Several important conclusions can be draw based on the preliminary experiment results. Firstly, the single-view biomarkers had distinct pathology patterns, and no single-view biomarkers were able to capture all the pathological changes. Secondly, the MRI and PET views could be clearly separated, and the views in the same modality could also form different clusters, each depicting a certain type of pathological changes. Thirdly, the different views had different strength in classification, and the clusters could provide a good reference of their strength. Fourthly, the combination of biomarkers could achieve much better results than the single-view biomarkers, and the inter-cluster biomarkers always gave the best results. Last but not least, the multi-view classification performance was primarily determined by the performance of individual views, but we could use the divergence to estimate the trade-off between the interference and synergy and predict the performance.

For the future work, we would include more subjects into our datasets and refine the current design of classifiers to convey more meaningful findings on AD / NC; MCI / NC; cMCI / ncMCI; AD / cMCI / ncMCI / NC. Current framework could only test two views at a time. We will extend this framework to accommodate multiple (greater than 2) views using the multivariate methods. Another future direction is that we will employ this cross-view pattern analysis framework to investigate cross-ROI synergies, since many ROIs have been repeatedly reported in previous multi-modal neuroimaging studies, i.e., the pattern of AD pathology start mainly in the hippocampus and entorhinal cortex, and subsequently spreads throughtout most of the temporal lobe and the posterior cingulate, finally reaches the parietal, prefrontal and orbitofrontal regions (Fan et al., 2008; Desikan et al., 2009; Risacher et al., 2009). It would also be interesting to incorporate other non-imaging features, such as ApoE genotype (Pastor and Goate, 2004) or CSF concentrations of *Aβ*_42_ (Motter et al., 1995) and tau (Vandermeeren et al., 1993). We will investigate their single-view and cross-view patterns.

## Author contributions

All authors listed, have made substantial, direct and intellectual contribution to the work, and approved it for publication.

### Conflict of interest statement

The authors declare that the research was conducted in the absence of any commercial or financial relationships that could be construed as a potential conflict of interest.
